# Enhancing energy predictions in multi-atom systems with multiscale topological learning†

**DOI:** 10.1039/d5ta02687c

**Published:** 2025-06-05

**Authors:** Dong Chen, Rui Wang, Guo-Wei Wei, Feng Pan

**Affiliations:** a School of Advanced Materials, Peking University, Shenzhen Graduate School Shenzhen 518055 China panfeng@pkusz.edu.cn; b Department of Mathematics, Michigan State University MI 48824 USA weig@msu.edu; c Simons Center for Computational Physical Chemistry, New York University New York NY 10003 USA; d Department of Electrical and Computer Engineering, Michigan State University MI 48824 USA; e Department of Biochemistry and Molecular Biology, Michigan State University MI 48824 USA

## Abstract

Lithium, a key element in high-energy-density batteries such as lithium-ion batteries, plays a crucial role in determining battery performance, safety, and longevity. Understanding how lithium atoms interact in clusters is essential for optimizing these properties. However, the complexity of these interactions grows exponentially as the number of Li atoms increases. While the rise of large models offers promising avenues for predictive accuracy in such multi-atom systems, the limited data available in material science hinders such breakthroughs. To answer the challenge, we present an interpretable topological learning framework designed to enhance the accuracy of energy predictions in multi-atom systems. This study explores the application of Persistent Topological Laplacians (PTLs), a multiscale topological method that effectively captures the intrinsic properties of many-body interactions. By applying PTLs, we offer a comprehensive analysis to uncover persistent topological features and geometric nuances in complex material systems. A dataset of 136 287 lithium clusters was analyzed using the proposed framework, and the results show that the PTL method aligns with traditional many-body theories, demonstrating its efficacy in capturing complex many-body interactions and improving prediction accuracy.

## Abstract 2

The multiscale topological learning framework, based on persistent topological Laplacians, captures complex interactions and enhances energy prediction accuracy in multi-atom systems.

## Graphical abstract

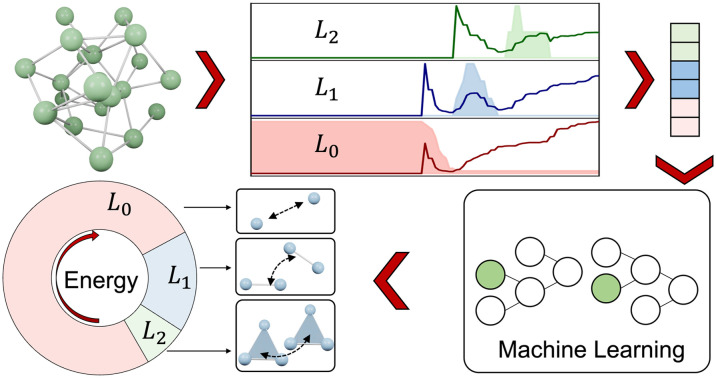

## 1 Introduction

In the realm of material science, understanding the behavior of multi-atom systems remains a fundamental yet challenging task, with the complexity of interactions increasing exponentially as the number of interacting particles grows.^1^ One of the most prominent examples is lithium, a key element in high-energy-density batteries like lithium-ion batteries, which plays a critical role in determining performance, safety, and longevity.^2^ Accurately predicting the energy and interactions within lithium clusters is crucial for advancing next-generation energy storage technologies.

However, for multi-atom systems, classical approaches, ranging from quantum chromodynamics in nuclear physics to quantum mechanics in atomic and molecular scales, often resort to reduced one- or two-body approximations.^3^ Higher-order perturbations, like those in Feynman diagrams and Ursell functions,^4,5^ are immensely valuable, but sometimes fall short in capturing non-perturbative effects. Similarly, statistical tools such as the BBGKY hierarchy^6^ provide critical insights into particle correlations but are often beset with formidable computational challenges. These traditional methods for studying such systems are often hindered by the sheer scale of the problem.

The rise of deep learning models, such as ChatGPT,^7,8^ has demonstrated the immense potential of machine learning in making accurate predictions based on vast amounts of data. These models excel at handling complex tasks in natural language processing by identifying intricate patterns and correlations across large datasets. Inspired by this success, machine learning has been applied to multi-atom systems to improve predictive performance in areas like energy calculations and structure prediction.^9,10^ However, two critical limitations hinder the applicability of large deep learning models in material science: the scarcity of data and the ‘black box’ nature of these models.^11,12^ First, the limited availability of high-quality experimental data in material science presents a major bottleneck. Gathering large-scale datasets can be prohibitively expensive and time consuming, limiting the effectiveness of deep models that rely on data richness to generalize across different systems.^13,14^ Without sufficient data, these models may fail to capture the intricate physical interactions at play in complex material systems like lithium clusters.^15^ Second, deep learning models, though effective at predictions, often lack interpretability, making it hard to understand the physical mechanisms behind their outcomes. In materials science, this is crucial for material discovery and design, where understanding behavior and properties is as important as accurate predictions.^16,17^

In the vast landscape of mathematical tools available, topological methods have emerged as powerful lenses through which various scientific disciplines perceive and understand intricate structures and interactions. The simplicial complex,^18^ for example, provides a topological framework for capturing interactions in multi-atom systems, while persistent homology has advanced our understanding in molecular^19^ and material science.^20–25^ And the Quotien Complex was recently introduced to study the inorangic system.^26^ In computational biology, differential geometry^27^ and algebraic graph^28^ theory shed light on the networks underlying life. Building on this, the persistent topological Laplacian (PTL) combines algebraic topology with topological spaces like simplicial complexes and manifolds, producing persistent spectral graph (PSG)^29^ and Hodge Laplacians,^30^ respectively. These methods link quantum mechanics, through zero-dimensional Hodge Laplacians, to topological spaces, promising new analytical tools for studying the many-body interactions with multi-atom systems. The reader is referred to a review.^31^

In this work, we propose a multiscale topological learning (MTL) framework, utilizing topological representations to reveal the intricate relationships of multi-atom systems, focusing on the Li clusters particularly. Drawing inspiration from algebraic topology, we introduce the PTL method, a novel approach designed to capture interactions inherent to multi-atom systems from a topological standpoint. This method allows the PTL to create a unified multiscale framework, adept at revealing topological persistence and distilling geometric shapes from intricate many-body interactions. As we navigate the bridge between the mathematical structures and the multi-atom systems, we harness the power of machine learning to validate our approach. Through rigorous qualitative and quantitative analyses of a diverse set of 136 287 Li cluster structures, spanning from 4-body to complex 40-body systems, we demonstrate the proficiency of the PTL in capturing and elucidating many-body interactions. Our findings underscore the topological method's capability to not only represent these interactions but also accurately predict properties intrinsic to multi-atom systems. This exploration, blending topological insights with physics, holds promise as a trailblazing framework, shedding light on the elaborate interactions that shape multi-atom systems and offering a fresh perspective on their study.

## 2 Results

In the realm of multi-atom systems, the intricacy of interactions poses a formidable challenge for traditional analytical techniques. To address this, we first introduce the simplicial complexes to represent structures, which provide a structured topological framework to encode many-body interactions. Also, drawing inspiration from the parallels between the Hodge Laplacian in algebraic topology and the kinetic operator in physics, we employ the PTL to facilitate a multiscale spectral analysis of physical systems. We show that the spectra of the PTL built from physical systems capture many-body interactions and reveal multifaceted physics.

**Fig. 1:**
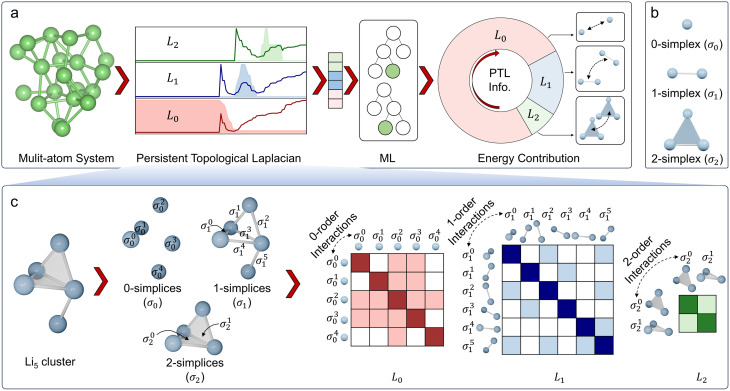
Overall scheme of multiscale topological learning to enhance the accuracy of energy prediction. (a) Workflow for energy prediction in multi-atom systems using the persistent topological Laplacian. The 20-atom Li cluster is treated as a simplicial complex, and the PTL method is applied to capture system characteristics across different dimensions, specifically the 0th, 1st, and 2nd dimensions, represented by *L*_0_, *L*_1_, and *L*_2_, respectively. Machine learning analysis reveals that the contribution of PTL information to energy prediction decreases with increasing dimensionality. Representative interactions for 0th, 1st, and 2nd orders are depicted on the right. (b) Illustration of 0-simplex, 1-simplex, and 2-simplex. (c) Demonstration of the schema for using topological Laplacians to capture the multi-order interactions within a Li_5_ cluster. The cluster is first expanded into 0, 1, and 2-simplex representations, and the corresponding topological Laplacians (*L*_0_, *L*_1_, and *L*_2_) are applied to record interactions of different orders.

The workflow of analyzing a multi-atom system using the PTL is illustrated in Fig. 1a. Specifically, the multi-atom system used in this work is a Li cluster system. There are 136 287 energy-paired Li cluster structures involved in the experiments, including 4-body, 5-body, 6-body, 7-body, 8-body, 9-body, 10-body, 20-body, and 40-body systems.^15^ The details and statistic information of all Li clusters are given in ESI Fig. S1†. With the PTL approach, multidimensional system information is transformed into features for the given structure. More precisely, the 0-, 1-, and 2-dimensional PTL features are generated for all the filtration parameters from 0.1 Å to 10 Å with an interval of 0.1 Å. Here, the upper bound of 10 Å was selected to prevent isolated atoms, ensuring all relevant interactions are captured. The lower bound of 0.1 Å allows for a fine-grained description of local interactions for Li-cluster system. These multi-dimensional features, acting as representative fingerprints of the many-body interactions, are then channeled into machine learning models to demonstrate their predictive power. When the many-body interactions are present in a multi-atom system, they subtly influence the PTL, creating nuanced deviations in the resulting features. As these features feed into the machine learning model, the prediction accuracy becomes an indirect gauge of these higher-order interactions' presence and impact. As shown in the final chart in Fig. 1a, the Laplacian matrices like *L*_0_, *L*_1_, and *L*_2_ embed the multi-order interactions of the system, representing interactions within vertices (0-simplices), edges (1-simplices), and triangles (2-simplices), respectively. Fig. 1b illustrates these 0, 1, and 2-simplices, which serve as fundamental building blocks in their respective dimensions. The quantitative results indicate that the contribution of features from each dimension of the PTL to energy prediction diminishes as the dimensionality increases, suggesting that while these higher-order interactions are complex and multifaceted, they introduce significant perturbations to the machine learning model's predictions. Fig. 1c illustrates an example of the schema for employing topological Laplacians to capture multi-order interactions within a Li_5_ cluster. The cluster is first expanded into 0-, 1-, and 2-dimensional spaces, corresponding to 0-, 1-, and 2-simplex topological spaces, and the associated topological Laplacian matrices (*L*_0_, *L*_1_, and *L*_2_) are applied to record interactions of various orders.

**Fig. 2:**
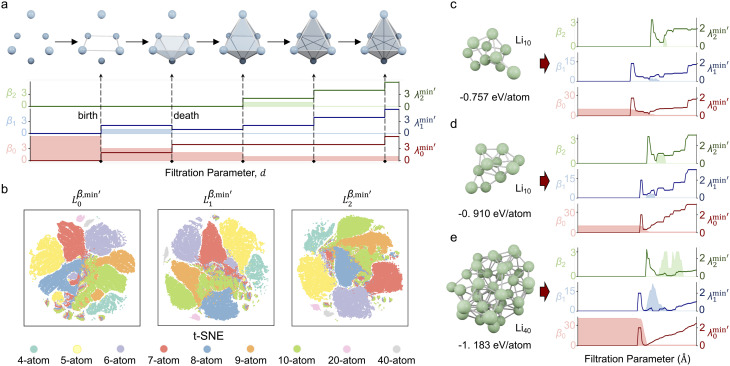
Persistent topological Laplacian. (a) Filtered simplicial complex along with the filtration parameter *d*. The filtration is considered in dimensions 0, 1, and 2. The lightly shaded parts indicate the values of the topological invariants in the different dimensions of the structure, *i.e.*, *β*_0_, *β*_1_, and *β*_3_, in the varying filtration parameters. The dark lines indicate the minimum values of the non-harmonic spectral information along with the changing filtration parameters in dimensions 0, 1, and 2. (
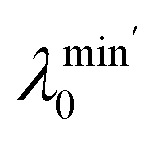
, 
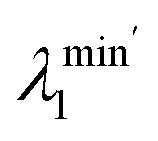
, and 
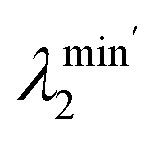
). (b) Two-dimension t-SNE embedding of the representation on PTL features. The colored points correspond to structures with different atomic numbers. More points of the same color clustered together, indicating a better clustering result. (c–e) PTL analysis for three specific structures. The structures in (c and d) contain 10 atoms each, but have binding energies of −0.757 eV per atom and −0.910 eV per atom, respectively. The structure in (e) contains 40 atoms and has richer PTL information, and its binding energy is −1.183 eV per atom.

We perform unsupervised cluster analysis on the dataset. The 0-, 1-, and 2-dimensional PTL features are denoted as 
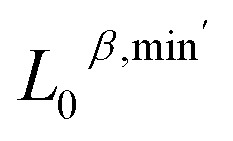
, 
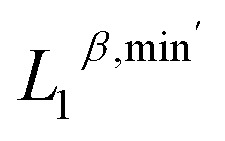
, and 
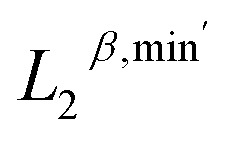
, respectively. Here, the superscript represents the harmonic part of the spectrum (*β*) and the minimum of the non-harmonic part of the spectrum, such as the smallest nonzero eigenvalues (min′). To investigate the impact of higher-dimensional PTL features on the system, we define three feature sets: (i) only 0-dimensional features 
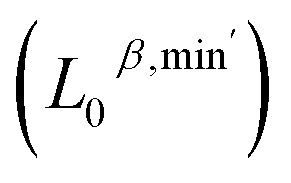
, (ii) both 0- and 1-dimensional features 
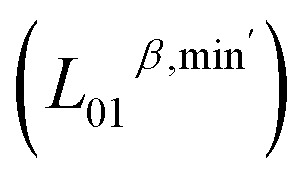
, and (iii) 0-, 1-, and 2-dimensional features 
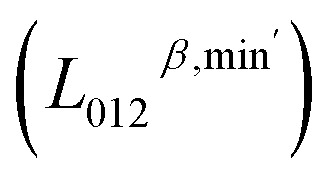
. Fig. 2b presents the two-dimensional t-SNE embedding of the representations for 
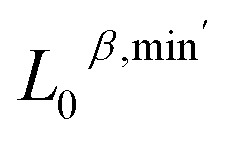
, 
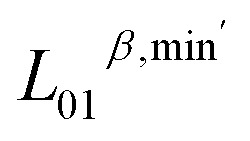
, and 
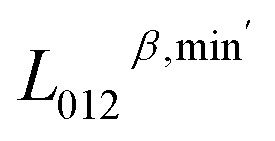
. The colored points in the figure represent structures with different atomic numbers. As shown in the figure, the clustering quality of the multi-atom system, reflected in the tendency of data points of the same color to group together, improves with the inclusion of high-dimensional information. However, the rate of improvement noticeably decreases, indicating that high-dimensional information contributes less to structure identification compared to low-dimensional information. A similar trend is observed when using Principal Component Analysis (PCA), a linear dimensionality reduction method, for visualization with the two largest principal components, as shown in Fig. S2.† Similarly, higher-order interactions are often treated as perturbations in many-body physics.^32^ In the clustering analysis, we only look at the clustering effect of each group feature to perform a qualitative analysis. The final results obtained are consistent with existing findings in many-body physics, which indicate that the PTL method can accurately capture the many-body interactions of the system.

Fig. 2c–e show the three structures analyzed by the PTL method, including two 10-particle systems (top, middle) and one 40-particle multi-atom system (bottom). For the systems of 10 particles, the structure's topological invariants *β*_1_, *β*_2_ in Fig. 2d contain a larger shaded area compared to Fig. 2c. It means that as the filtration parameter increases, the Li cluster in Fig. 2d has more 1- and 2-dimensional cavities. Note the topological cavities here are analogous to the many-body interactions within the system. The binding energies of structure in Fig. 2c and d are −0.756 eV per atom and −0.910 eV per atom, which implies that more many-body interactions favor the stability of the system. As for the non-harmonic information 
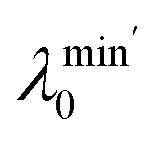
, 
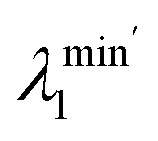
, and 
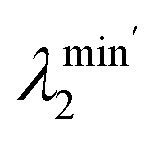
, the lines in Fig. 2d also enclose more area, which means that the particles in Fig. 2d have more complex connectivity relationships. Fig. 2e shows a 40-atom lithium cluster, which contains more high-dimensional topological and geometric complexity (*β*_1_ and *β*_2_, 
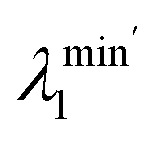
 and 
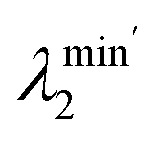
) than aforementioned two 10-atom lithium clusters do and has a lower binding energy of −1.183 eV per atom. In addition, we generated topological fingerprints of the structures using the persistent homology method, which is equivalent to the features from the harmonic spectra part in the PTL method, for the three structures mentioned above, as shown in ESI Fig. S3.†

**Fig. 3:**
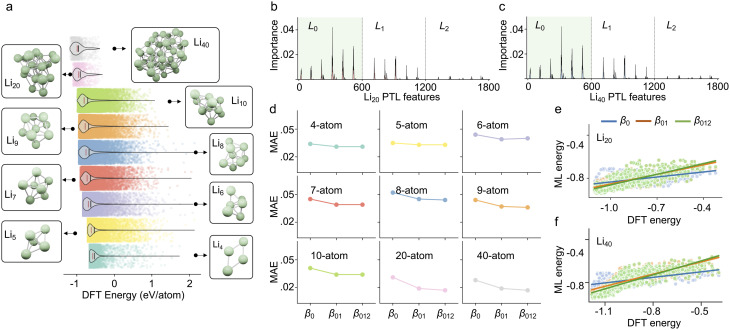
Results analysis. (a) Energy distribution of multi-atom systems containing different numbers of atoms. As the number of atoms increases, the energy (eV per atom) of the system gradually decreases. The red line is the median energy, and the black line is the mean energy (see Fig. S1†). (b) The MAE of cross-validation for multi-atom systems with different numbers of atoms using different topological information. *β*_0_ means only 0-dimensional topological information is used. *β*_01_ means both *β*_0_ and *β*_1_ are used. *β*_012_ means all *β*_0_, *β*_1_ and *β*_2_ are used in the prediction. (c and d) Comparison between MTL-based prediction results and DFT results of the formation energy of Li_20_ and Li_40_. (e) The RMSE of the machine learning prediction results for Li_20_, and Li_40_ structures using different topological features.

Fig. 3a demonstrates the binding energy distribution of all 136 287 Li cluster structures, from bottom to top, which are Li_4_ to Li_10_ systems, Li_20_ system, and Li_40_ system, respectively. It can be seen that the average binding energy per atom of each type of system gradually decreases with the increase of the number of particles in the system, which indicates that the complex interactions in the multi-particle system enhance the stability of the system. The mean and median energies of all structures can be found in ESI Fig. S1.† To better understand how different dimensional PTL features contribute to the multi-atom system, we first perform a feature analysis of the PTL features. Specifically, to explore the Laplacian spectral information, we extract six key properties from each dimension's Laplacian matrix: the multiplicity of zero eigenvalues (*β*), the minimum nonzero eigenvalue (min′), the maximum, the mean, the standard deviation of the eigenvalues, and the generalized mean graph energy.^33–35^ Consequently, six values are used per Laplacian at each dimension and filtration scale. Given a multiscale range from 0.1 Å to 10 Å with a step size of 0.1 Å each dimension contributes 600 features, resulting in a total of 1800 features across the three considered dimensions. The distribution of feature importance for predicting Li_20_ and Li_40_ is shown in Fig. 3b and c. It is observed that 0-dimensional features contribute the most to the system, followed by 1-dimensional features, while 2-dimensional features have a certain but relatively minor contribution. This trend aligns with the qualitative analysis in Fig. 2b. Additionally, the feature importance were extracted from gradient-boosted decision trees (GBDT) models trained exclusively on 4- to 10-atom Li clusters, meaning that Li_20_ and Li_40_ clusters were unseen during training. Detailed results can be found in ESI Table S2.† Interestingly, using only the *L*_0_ features yields the best predictive performance for Li_20_ and Li_40_, whereas models incorporating *L*_01_ and *L*_012_ perform relatively worse. This may be due to the inclusion of additional feature dimensions leading to overfitting in the same model setting, particularly when predicting structurally distinct systems such as the unseen Li_20_ and Li_40_ multi-atom clusters.

To avoid the overfitting issues, we further perform supervised learning only using the harmonic spectral features of the PTL to explore the contribution of high-dimensional information to energy prediction. We set up three sets of features, *i.e.*, *β*_0_ containing only 0-dimensional topological information, *β*_01_ containing 0- and 1-dimensional topological information, and *β*_012_ containing 0-, 1-, and 2-dimensional topological information. The RMSE, MAE, and Pearson correlation coefficient (PCC) are used as evaluation metrics, and their definitions are given in ESI Note S1.† Subsequently, for each system, cross-validation is performed for each of these three sets of features. The GBDT algorithm was employed as the regressor for cross-validation, utilizing 1D PTL features as input. Additionally, PTLs can generate image-like features,^36^ which are suitable for models like CNNs or Transformers that process image-like inputs. Only one parameter set is used in all machine learning processes, as detailed in ESI Note S1.† The results are shown in Fig. 3d. For all types of systems, the MAE of prediction decreases while adding higher dimensional topological information. However, the improvement of prediction accuracy diminishes gradually, indicating that the higher dimensional information contributes less to the prediction accuracy. In addition, we found that the MAE is lower for systems with more atoms, *i.e.*, 20-atom and 40-atom compared to other systems. It indicates that as the number of particles in the system increases, the system will have more higher-order interactions within the system, and the PTL method can capture these higher-order interactions, thus increasing the accuracy of the model prediction. It is also consistent with the previous analysis of the special cases in Fig. 2c–e, indicating that as the number of particles in the system increases, the multi-atom system will contain richer high-order interactions. Furthermore, we trained models separately using *β*_1_, *β*_2_, and *β*_12_. The results were worse compared to those incorporating *β*_0_, highlighting the primary contribution of low-dimensional information and low-order interactions. The cross-validation results for all types of cluster systems are listed in Table S4.† All cross-validation experiments were carried out ten times using different random seeds. The final results were reported using the average of the ten experiments.

Furthermore, we explored the contribution of high-dimensional structural features to the ranking power of the multi-atom systems. The ranking power of the model can be used to find the lowest energy structural configurations. Specifically, we trained a machine learning model using all Li_4_–Li_10_ data, and subsequently, used such a model to predict the structural energy of Li_20_ and Li_40_. To compare the ranking power, the PCC is used to evaluate the model. Fig. 3e shows the comparison between machine learning prediction results and DFT calculation results of the binding energy of Li_20_. The best-ranking power (PCC = 0.771) is obtained by using *β*_012_, while the ranking power for *β*_0_ and *β*_01_ are 0.508 and 0.742, respectively. We also tested the performance by using *β*_1_, *β*_2_, and *β*_12_, as listed in Table S3,† which were shown worse performances. For *β*_0_, adding features of *β*_1_ information can improve the prediction accuracy by 46.1%. Although *β*_012_ contains information of 0-, 1-, and 2-dimension spaces, the prediction accuracy is only 4.0% better compared to *β*_01_. As shown in Fig. 3f, similar results can also be found for Li_40_. The ranking power for *β*_0_, *β*_01_, and *β*_012_ are 0.592, 0.801, and 0.817. The improvement of *β*_01_ for *β*_0_ is 35.3%, while the improvement of *β*_012_ for *β*_01_ is only 2.0%. By adding high-dimensional information, the prediction accuracy of the model continues to improve, but the added higher-dimensional information has only a smaller contribution. Our results indicate that while high-dimensional information enhances prediction accuracy, its contribution gradually diminishes as dimensionality increases. Similarly, the influence of many-body interactions on approximation decreases with higher-order interactions. Models trained using only *β*_1_, *β*_2_, or *β*_12_ performed worse compared to those incorporating *β*_0_, further emphasizing the significance of lower-dimensional features. The prediction results using the harmonic part of the Laplacian, evaluated with RMSE, MAE, and PCC, are summarized in Table S3.† The machine learning processes in this work were repeated 10 times and the average results are used in the final demonstration.

## 3 Discussion

In this work, we explore the intricate relationship between many-body interactions in lithium clusters and the corresponding simplicial complex structures across various dimensions. This mapping allows us to introduce the combinatorial Laplacian operator, akin to the discretized energy operator in physics, offering a new perspective for analyzing material structures. Through the PTL method, we generate a series of combinatorial Laplacians, revealing harmonic and non-harmonic spectra that encapsulate essential topological and geometric features of the multi-atom system, such as Li cluster.

By leveraging these spectra through the PTL method, multi-dimensional features emerge, capturing complex many-body interactions at various scales. When integrated with machine learning models, these PTL-based features reveal the subtleties of higher-order interactions, reflected as perturbations in the model's predictive power. In this study, 136 287 Li cluster structures, ranging from 5-atom to 40-atom systems, were analyzed to validate the proposed PTL-based topological learning scheme. The results of clustering experiments demonstrated that PTL-based features provide strong clustering performance, with high-dimensional information contributing positively to clustering, though its effect diminishes with increasing dimensionality.

Further validation was conducted by categorizing the data into nine groups based on the number of atoms in the system and performing cross-validation. The cross-validation results reaffirmed that while high-dimensional features enhance prediction accuracy, their contribution diminishes with increasing dimensionality. For larger systems with complex many-body interactions, such as Li_20_ and Li_40_, the PTL model effectively ranked these systems by energy, demonstrating that lower-dimensional features are more influential in improving prediction accuracy. Additionally, a comparison was made between the Li_40_ prediction results and those obtained from a previous persistent homology-based method, which was used to identify stable configurations of Li_40_.^15^ The latter method reported a PCC of 0.95, while the proposed method in this study achieved a PCC of 0.968 (without any parameter tuning, as shown in Table S2†).

The proposed multiscale topological learning scheme excels at capturing interactions across multiple orders in Li clusters, approximating the system's intrinsic properties with remarkable accuracy. Experimental results using Li clusters underscore the alignment of this approach with traditional many-body theory, reinforcing its robustness and precision in predicting system energy. Beyond lithium clustering studies, this framework demonstrates significant potential across various fields. In materials science, PTLs can be used to encode materials into a topological space, enabling material discovery within a more manageable, smaller topological space. This not only streamlines the design process but also accelerates the discovery of new materials, enhancing the efficiency of material development. In catalysis, the PTL method effectively models and predicts the unique configurations formed between catalytic surfaces and catalysts. By accurately capturing these configurations, it accelerates the design and optimization of catalytic materials, which is essential for advancing catalytic processes and developing novel catalytic systems. As for the molecular and biological sciences, PTLs can be applied to model molecular systems and interactions within complex environments, such as drug–drug complexes, protein–ligand interactions, and protein–protein systems. Traditional molecular dynamics simulations often face challenges when dealing with large systems, but PTL serves as a promising computational tool for extracting higher-order information. This approach provides more accurate predictions of molecular interactions, offering deeper insights into the complex dynamics of biological systems. As such, PTL holds considerable promise for applications in drug discovery, protein engineering, and bioinformatics.

## 4 Methods

In this section, we introduce some key principles from classical many-body theories and algebraic topology. We will briefly discuss foundational concepts including simplices, simplicial complexes, and the boundary operator, and then delve deeper into homology, persistent homology, and the persistent topological Laplacian.

### 4.1 Reduced density operator and higher-order interactions

**Fig. 4:**
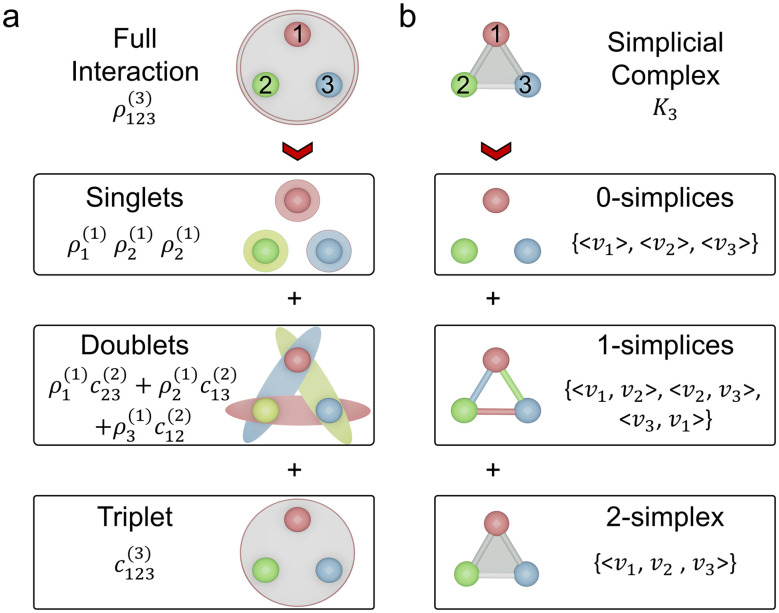
Illustration of the interaction expansion for the three-body system, and the simplicial complex for the three-point system. (a) The full interaction of the three-body system represents by the density operator *ρ*_123_^(3)^, which can be composed of singlets (*ρ*_1_^(1)^, *ρ*_2_^(1)^, and *ρ*_3_^(1)^), doublets (*ρ*_1_^(1)^*c*_23_^(2)^, *ρ*_2_^(1)^*c*_13_^(2)^, and *ρ*_3_^(1)^*c*_12_^(2)^), and triplet (*c*_123_^(3)^). Each colored sphere corresponds to the one particle operator and the colored circles/ellipses to the correlations. (b) The simplicial complex, *K*_3_, is the combination of 0-simplices (〈*v*_1_〉, 〈*v*_2_〉, and 〈*v*_3_〉), 1-simplices (〈*v*_1_, *v*_2_〉, 〈*v*_2_, *v*_3_〉, and 〈*v*_3_, *v*_1_〉), and the 2-simplex (〈*v*_1_, *v*_2_, *v*_3_〉). The number in the sphere corresponds to the subscript in the operator/vertex.

In many-body systems, the reduced density operator (RDO) helps capture interactions between a subset of particles without needing to consider the entire system. For an *N*-particle system, the *n*-particle RDO, *ρ*^(*n*)^, provides a view of the interactions among *n* particles while marginalizing out the remaining *N* − *n* particles.^37^ A key insight from RDOs is the ability to break down complex, higher-order interactions into contributions from lower-order ones. As an example in Fig. 4a, consider a three-particle system described by *ρ*_123_^(3)^, which can be decomposed as: *ρ*_123_^(3)^ = *ρ*_1_^(1)^*ρ*_2_^(1)^*ρ*_3_^(1)^ + *ρ*_1_^(1)^*c*_23_^(2)^ + *ρ*_2_^(1)^*c*_13_^(2)^ + *ρ*_3_^(1)^*c*_12_^(2)^ + *c*_123_^(3)^, where the correlation function *c*^(*n*)^, *n* = 1, 2, 3, measures the degree of correlation among *n* particles. This hierarchical representation mirrors the way simplicial complexes in topology use simplices of different dimensions to represent interactions within a structure. Lower-order interactions, like pairwise correlations, are often dominant and easier to compute, while higher-order terms (three or more particles) capture more nuanced relationships and can be treated as perturbative corrections.^4,32^ This hierarchical decomposition allows us to focus on the essential structure of interactions while systematically including higher-order effects.

### 4.2 Simplex and simplicial complex

Topologically, a simplex is a fundamental building block that generalizes points, line segments, triangles, tetrahedron, and higher-dimensional analogs (Fig. 1b and S4a†). A *k*-simplex is the convex hull of *k* + 1 affinely positioned vertices, denoted as *σ*_*k*_ = 〈*v*_0_, *v*_1_, …, *v*_*k*_〉. A simplicial complex, denoted as *K*, is formed by assembling simplices such that every face of a simplex is also in the complex, encapsulating their spatial relationships. The Vietoris–Rips complex connects points within a specified distance, evolving to reveal topological features at different scales (Fig. S4d†). Within such complexes, cycles (*e.g.*, 0-cycle, 1-cycle, 2-cycle) highlight topological attributes like gaps or cavities (Fig. S4c†). Simplicial complexes are versatile tools for depicting and analyzing complex systems. In multi-atom systems, each simplex represents interacting particles, with its dimensionality indicating the number of bodies involved. For instance, Fig. 4b shows a simplicial complex of a 3-body system, *K*_3_, containing three 0-simplices (〈*v*_1_〉, 〈*v*_2_〉, 〈*v*_3_〉), three 1-simplices (〈*v*_1_, *v*_2_〉, 〈*v*_2_, *v*_3_〉, 〈*v*_3_, *v*_1_〉), and one 2-simplex (〈*v*_1_, *v*_2_, *v*_3_〉). This representation shows a one-to-one correspondence between the reduced density operator *ρ* and simplex *σ*, providing a geometrically and topologically insightful framework to understand multi-atom systems.

### 4.3 Boundary operator and chain complex

With the foundations of simplices and simplicial complexes established, we turn to the hierarchical and topological aspects. *k*-chains are formal combinations of *k*-simplices, which can be algebraically combined to form chain groups denoted as *C*_*k*_(*K*). Here, these chains are considered under modulo two operations, 
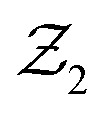
. The boundary operator *∂*_*k*_: *C*_*k*_(*K*) → *C*_*k*−1_(*K*) maps a *k*-simplex to its (*k* − 1)-dimensional faces. For example, applying *∂*_*k*_ to a 2-simplex (triangle) yields its three 1-simplices (edges). Fig. S4b† illustrates how *∂*_*k*_ operates from dimension 0 to 3. The matrix representation of *∂*_*k*_ is denoted 
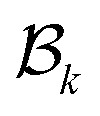
, as shown in ESI Fig. S1.†

A chain complex is a sequence of chain groups connected by boundary operators:1
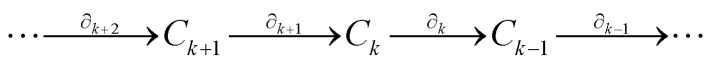


This structure ensures continuity, with a key property: applying a boundary operator twice yields zero, *i.e.*, *∂*_*k*_*∂*_*k*+1_ = 0. The adjoint boundary operator 
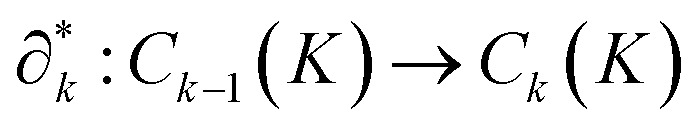
 acts in the reverse direction, increasing the dimension of simplices. Its matrix representation, 
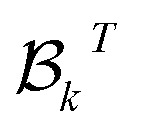
, is the transpose of 
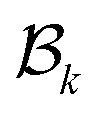
.

### 4.4 Laplacian and spectrum analysis

The combinatorial Laplacian is a key tool in discrete geometry and algebraic topology, extending the concept of the graph Laplacian to higher dimensions. It provides insights into the structure of simplicial complexes, similar to how the graph Laplacian reveals connectivity in graphs. For a graph viewed as a 1-dimensional complex, the Laplacian matrix is 
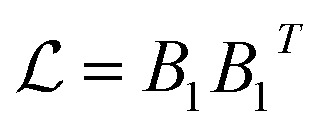
, where *B*_1_ is the boundary matrix. This generalizes to higher dimensions with the Laplacian defined as:2
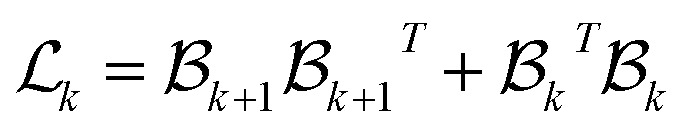
where 
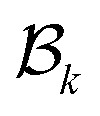
 represents the boundary operator for *k*-simplices. The term 
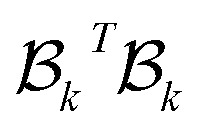
 accounts for connectivity among *k*-simplices, while 
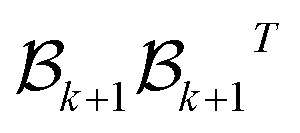
 captures interactions involving (*k* + 1)-simplices.

In chain complexes, the combinatorial Laplacian *δ*_*k*_ is defined as:3
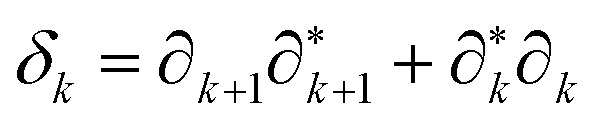
where *∂*_*k*_ and 
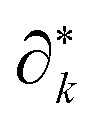
 are boundary operators and their adjoints.

The topological Laplacian extends the graph Laplacian to higher-dimensional simplicial complexes, with eigenvalues revealing topological and geometric properties. It is positive semidefinite, meaning all eigenvalues are non-negative. Zero eigenvalues correspond to topological invariants such as Betti numbers (*β*_*k*_), which count independent components,^38^ cycles, and cavities. The smallest non-zero eigenvalue, or spectral gap (*λ*^min^_*k*_), reflects the geometric connectivity of the complex. This analysis uses zero multiplicities and the smallest positive eigenvalues to elucidate topological and geometric features.

### 4.5 Persistent topological Laplacians

Persistent topological Laplacians, or multiscale topological Laplacians, arise from research in both differential manifolds^30^ and discrete point clouds.^29^ Central to persistent topological Laplacians^29,34,39,40^ and persistent homology^41,42^ is the concept of filtration, which allows for multiscale analysis. Filtration is parametrized by a scale *d*, adapting to the data structure under study. For instance, in a distance set, edges are added between vertices if their distance is below a cutoff value. Increasing this cutoff generates a sequence of nested graphs, where each graph at a lower cutoff is a subset of those at higher cutoffs (Fig. 2a). Similar nested simplicial complexes can be created using the Vietoris–Rips, Čech, and alpha complexes. This study focuses on the Vietoris–Rips complex.

Mathematically, these nested simplicial complexes are represented as follows:

4 *∅* ⊆ *K*_*d*_0__ ⊆ *K*_*d*_1__ ⊆ ⋯ ⊆ *K*_*d*_*n*__ = *K*

Here, for any two values *d*_*i*_ < *d*_*j*_, the complex *K*_*d*_*i*__ is a subset of *K*_*d*_*j*__. A chain complex associated with a specific filtration step consists of a sequence of Abelian groups (or modules) connected by boundary homomorphisms, which can be represented as follows:5
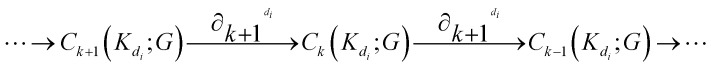
where *C*_*k*_(*K*_*d*_*i*__; *G*) denotes the chain group in the *k*-dimensional space at the specific filtration step *d*_*i*_. Define *C*_*k*+1_^*a*,*b*^ as the set containing elements *x* in *C*_*k*+1_^*b*^ such that the boundary operator ∂_*k*+1_^*b*^ applied to *x* yields an element in *C*_*k*_^*a*^. Formally, this is expressed as *C*_*k*+1_^*a*,*b*^ = {*x* ∈ *C*_*k*+1_^*b*^∣∂_*k*+1_^*b*^*x* ∈ *C*_*k*_^*a*^}.

The persistent boundary operator, denoted as ∂_*k*+1_^*a*,*b*^, maps from *C*_*k*+1_^*a*,*b*^ to *C*_*k*_^*a*^ and is defined by the action ∂_*k*+1_^*a*,*b*^*x* = ∂_*k*+1_^*b*^*x* for any *x* in *C*_*k*+1_^*a*,*b*^. The framework can be expressed by:6
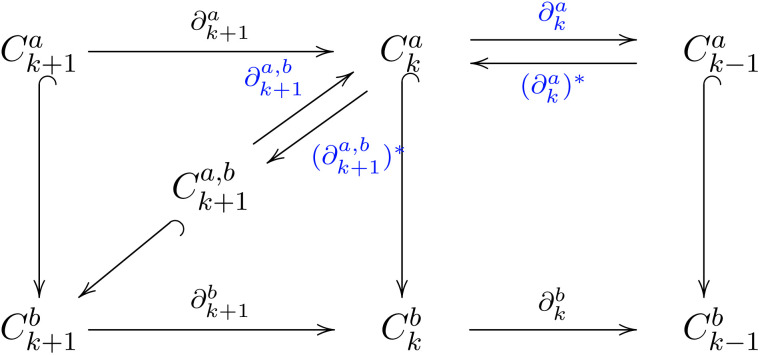


The *k*-th persistent topological Laplacian is defined as

7 *δ*_*k*_^*a*,*b*^ = ∂_*k*+1_^*a*,*b*^ ∘ (∂_*k*+1_^*a*,*b*^)* + (∂_*k*_^*a*^)* ∘ ∂_*k*_^*a*^

Its harmonic part, ker*δ*_*k*_^*a*,*b*^, corresponds to the (*a*, *b*)-persistent homology *H*_*k*_^*a*,*b*^ = im(*H*_*k*_(C_*_^a^) → *H*_*k*_(C_*_^b^)),^43^ encoding persistent homology information. Spectral analysis of the Laplacian matrices for each *∂*_*k*_ and *∂*_*k*+1_ provides insights into topological and geometric attributes at different scales. Fig. 2a shows persistent topological Laplacian analysis for a system of six particles, with varying *β* values and spectral data indicating changes in connectivity and geometric structure.

## Code availability

The source code for the persistent topological Laplacian analysis, implemented in Python, is publicly available in the GitHub repository at https://github.com/ChenDdon/LiCluster.

## Author contributions

Dong Chen designed the project, performed computational studies, analyzed data, wrote the first draft, and revised the manuscript. Rui Wang drafted part of the method. Guo-Wei Wei conceptualized and supervised the project, revised the manuscript, and acquired funding. Feng Pan supervised the project and acquired funding.

## Conflicts of interest

The authors declare no competing interests.

## Supplementary Material

TA-013-D5TA02687C-s001

## Data Availability

The cluster structures and the energy data are publicly available at https://github.com/ChenDdon/LiCluster.

## References

[cit1] Stillinger F. H. (1999). Exponential multiplicity of inherent structures. Phys. Rev. E: Stat. Phys., Plasmas, Fluids, Relat. Interdiscip. Top..

[cit2] Scrosati B., Garche J. (2010). Lithium batteries: Status, prospects and future. J. Power Sources.

[cit3] Parr R. G., Gadre S. R., Bartolotti L. J. (1979). Local density functional theory of atoms and molecules. Proc. Natl. Acad. Sci. U. S. A..

[cit4] Wei G. W., Snider R. F. (1996). Discrete basis representation of ursell operators. Phys. Rev. E: Stat. Phys., Plasmas, Fluids, Relat. Interdiscip. Top..

[cit5] Ursell H. D. (1927). The evaluation of gibbs' phase-integral for imperfect gases. Math. Proc. Cambridge Philos. Soc..

[cit6] Bogoliubov N. N. (1946). Problems of dynamical theory in statistical physics (gostekhisdat, moscow, 1946)[in russian]; nn bogoliubov. J. Phys..

[cit7] Vaswani A. , Shazeer N., Parmar N., Uszkoreit J., Jones L., Gomez A. N., Kaiser Ł. and Polosukhin I., Attention is all you need, Advances in Neural Information Processing Systems, 2017, vol. 30

[cit8] Ouyang L., Wu J., Jiang X., Almeida D., Wainwright C., Mishkin P., Zhang C., Agarwal S., Slama K., Ray A. (2022). *et al.*, Training language models to follow instructions with human feedback. Adv. Neural Inf. Process. Syst..

[cit9] Reiser P., Neubert M., Eberhard A., Torresi L., Zhou C., Shao C., Metni H., van Hoesel C., Schopmans H., Sommer T. (2022). *et al.*, Graph neural networks for materials science and chemistry. Commun. Mater..

[cit10] Wei J., Chu X., Sun X.-Y., Xu K., Deng H.-X., Chen J., Wei Z., Lei M. (2019). Machine learning in materials science. InfoMat.

[cit11] Rodrigues J. F., Florea L., de Oliveira M. C. F., Diamond D., Oliveira O. N. (2021). Big data and machine learning for materials science. Discover Mater..

[cit12] Sutton C., Boley M., Ghiringhelli L. M., Rupp M., Vreeken J., Scheffler M. (2020). Identifying domains of applicability of machine learning models for materials science. Nat. Commun..

[cit13] Zhang Y., Chen L. (2018). A strategy to apply machine learning to small datasets in materials science. npj Comput. Mater..

[cit14] Cai J., Chu X., Xu K., Li H., Wei J. (2020). Machine learning-driven new material discovery. Nanoscale Adv..

[cit15] Chen X., Chen D., Weng M., Jiang Y., Wei G.-W., Pan F. (2020). Topology-based machine learning strategy for cluster structure prediction. J. Phys. Chem. Lett..

[cit16] Zhong X., Gallagher B., Liu S., Kailkhura B., Hiszpanski A., Han T. Y.-J. (2022). Explainable machine learning in materials science. npj Comput. Mater..

[cit17] Faraji Niri M., Reynolds C., Román Ramírez L. A. A., Kendrick E., Marco J. (2022). Systematic analysis of the impact of slurry coating on manufacture of li-ion battery electrodes *via* explainable machine learning. Energy Storage Mater..

[cit18] Edwin H. S. and Henry Spanier E., Algebraic Topology, Springer Science & Business Media, 1989

[cit19] Chen D., Zhang M.-Z., Chen H.-B., Xie Z.-W., Guo-Wei W., Pan F. (2020). Persistent homology for the quantitative analysis of the structure and stability of carboranes. Chin. J. Struct. Chem..

[cit20] Carlsson G. (2009). Topology and data. Bull. Am. Math. Soc..

[cit21] Jacob T., Micucci C. P., Hymel J. H., Maroulas V., Vogiatzis K. D. (2020). Representation of molecular structures with persistent homology for machine learning applications in chemistry. Nat. Commun..

[cit22] Lee Y., Barthel S. D., Dłotko P., Mohamad Moosavi S., Hess K., Smit B. (2017). Quantifying similarity of pore-geometry in nanoporous materials. Nat. Commun..

[cit23] Hiraoka Y., Nakamura T., Hirata A., Escolar E. G., Matsue K., Nishiura Y. (2016). Hierarchical structures of amorphous solids characterized by persistent homology. Proc. Natl. Acad. Sci. U. S. A..

[cit24] Lee Y., Barthel S. D., Dłotko P., Moosavi S. M., Hess K., Smit B. (2018). High-throughput screening approach for nanoporous materials genome using topological data analysis: application to zeolites. J. Chem. Theor. Comput..

[cit25] Anand D. V., Xu Q., Wee J. J., Xia K., Sum T. C. (2022). Topological feature engineering for machine learning based halide perovskite materials design. npj Comput. Mater..

[cit26] Hu C.-S., Mayengbam R., Xia K., Chien Sum T. (2025). Quotient complex (qc)-based machine learning for 2d hybrid perovskite design. J. Chem. Inf. Model..

[cit27] Nguyen D. D., Wei G.-W. (2019). DG-GL: Differential geometry-based geometric learning of molecular datasets. Int. J. Numer. Methods Biomed. Eng..

[cit28] Nguyen D. D., Wei G.-W. (2019). AGL-score: algebraic graph learning score for protein–ligand binding scoring, ranking, docking, and screening. J. Chem. Inf. Model..

[cit29] Wang R., Nguyen D. D., Wei G.-W. (2020). Persistent spectral graph. Int. J. Numer. Methods Biomed. Eng..

[cit30] Chen J., Zhao R., Tong Y., Wei G.-W. (2021). Evolutionary de rham-hodge method. Dyn. Contin. Discret. Impuls. Syst. Ser. B.

[cit31] Xiaoqi W., Wei G.-W. (2025). Persistent topological Laplacians–a Survey. Found. Data Sci..

[cit32] Liboff R. L., Perona G. E. (1967). Compatibility requirements in bbgky expansion. J. Math. Phys..

[cit33] Wee J. J., Xia K. (2022). Persistent spectral based ensemble learning (PerSpect-EL) for protein–protein binding affinity prediction. Briefings Bioinf..

[cit34] Meng Z., Xia K. (2021). Persistent spectral–based machine learning (PerSpect ML) for protein-ligand binding affinity prediction. Sci. Adv..

[cit35] Liu X., Feng H., Wu J., Xia K. (2021). Persistent spectral hypergraph based machine learning (PSH-ML) for protein-ligand binding affinity prediction. Briefings Bioinf..

[cit36] Jiang P., Chi Y., Li X.-S., Meng Z., Liu X., Hua X.-S., Xia K. (2022). Molecular persistent spectral image (mol-psi) representation for machine learning models in drug design. Briefings Bioinf..

[cit37] Alavi S., Wei G. W., Snider R. F. (1998). Chain relations of reduced distribution functions and their associated correlation functions. J. Chem. Phys..

[cit38] Eckmann B. (1944). Harmonische funktionen und randwertaufgaben in einem komplex. Comment. Math. Helvetici.

[cit39] Chen D., Liu J., Wu J., Wei G.-W. (2023). Persistent hyperdigraph homology and persistent hyperdigraph Laplacians. Foundations of Data Science.

[cit40] Mémoli F., Wan Z., Wang Y. (2022). Persistent laplacians: Properties, algorithms and implications. SIAM J. Math. Data Sci..

[cit41] Edelsbrunner H. , Letscher D., and Zomorodian A., Topological persistence and simplification, in Proceedings 41st Annual Symposium on Foundations of Computer Science, IEEE, 2000, pp. 454–463

[cit42] Zomorodian A., Carlsson G. (2005). Computing persistent homology. Discrete Comput. Geom..

[cit43] Liu J., Li J., Wu J. (2024). The algebraic stability for persistent laplacians. Homol. Homotopy Appl..

