# Supplementary material for: Enhancing energy predictions in multi-atom systems with multiscale topological learning
Source: J Mater Chem A Mater. 2025 Jun 5;13(27):21555–63. doi: 10.1039/d5ta02687c (PMC12153608; doi:10.1039/d5ta02687c)
Supplement: TA-013-D5TA02687C-s001 [file TA-013-D5TA02687C-s001.pdf]

Supplementary Information (SI) for Journal of Materials Chemistry A.
This journal is © The Royal Society of Chemistry 2025

Supplementary Information
Enhancing Energy Predictions in Multi-Atom Systems with Multiscale Topological Learning

Dong Chen[1,2], Rui Wang *[2], Guo-Wei Wei †[2,3,4] and Feng Pan ‡[1]

[1] *School of Advanced Materials, Peking University, Shenzhen Graduate School, Shenzhen 518055, China*
[2] *Department of Mathematics, Michigan State University, MI, 48824, USA*
[3] *Department of Electrical and Computer Engineering, Michigan State University, MI 48824, USA*
[4] *Department of Biochemistry and Molecular Biology, Michigan State University, MI 48824, USA*

This document contains supplementary information which were not necessary to include in the central part of the paper but might be of interest to readers. This supplementary material includes the following sections:

Contents

S1 Supplementary Note 1 2

S2 Supplementary Note 2: Figures 3

S3 Supplementary Note 3: Tables 5

*Current address: Simons Center for Computational Physical Chemistry, New York University, New York, NY, 10003
†Corresponding author: weig@msu.edu
‡Corresponding author: panfeng@pkusz.edu.cn

S1 Supplementary Note 1

Evaluation Metrics In this study, the Pearson correlation coefficient (PCC) is used in the energy prediction, and it is defined as below:

$$PCC = \frac{\sum (x_i - \bar{x})(y_i - \bar{y})}{\sqrt{\sum (x_i - \bar{x})^2 \sum (y_i - \bar{y})^2}} \tag{1}$$

where x_i is the value of the x variable in ith sample, $\bar{x}$ is the mean of the values of the x variable, y_i is the value of the y variable in the ith sample, $\bar{y}$ is mean of the values of the y variable. The PCC explains the relationship between the x variable and y variable.

The root mean squared error (RMSE) is defined as below:

$$\text{RMSE} = \sqrt{\frac{1}{n}\sum_{i=1}^{n}(y_i - \hat{y_i})^2} \tag{2}$$

where y_i and $\hat{y_i}$ are predicted value and true value of ith sample respectively.

The mean absolute error (MAE) measures the mean difference between the prediction and the true value,

$$\text{MAE} = \frac{1}{n}\sum_{i=1}^{n}|y_i - \hat{y_i}| \tag{3}$$

where y_i and $\hat{y_i}$ are predicted value and true value of ith sample respectively.

GBDT parameters. In the machine learning task, we use the gradient boosted decision trees (GBDT) algorithm to predict the multi-atom system's energy. The 'n_estimators' is setting to 15000, 'max_depth' is setting to 7, 'min_samples_split' is setting to 5, 0.8 of the subsample is used, and the learning rate of the model is setting to 0.001. All other parameters were using the default values in the algorithm.[1]

S2 Supplementary Note 2: Figures

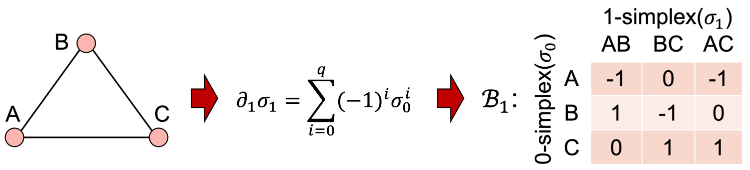

Supplementary Figure S1: Illustration of the matrix representation of the boundary operator.

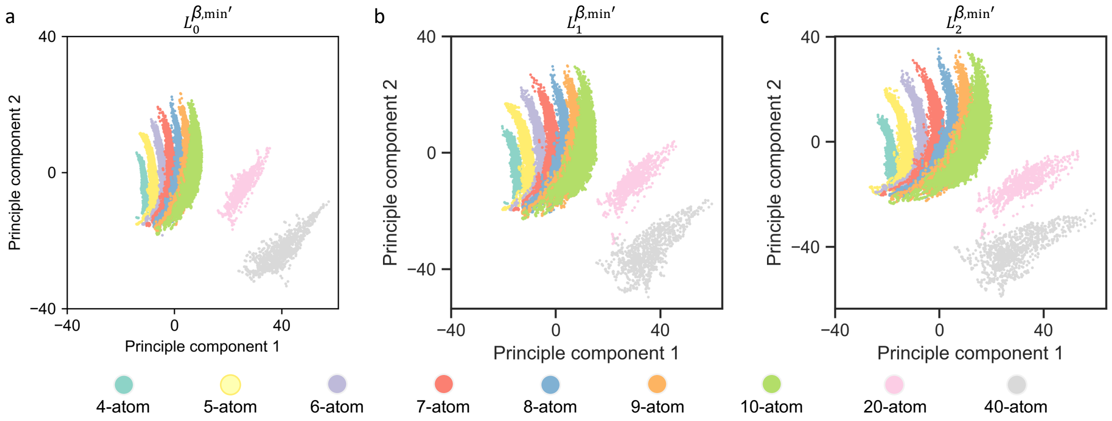

Supplementary Figure S2: Two-dimension PCA embedding of the representation on PLT features. The colored points correspond to structures with different atomic numbers. More points of the same color clustered together, indicating a better clustering result.

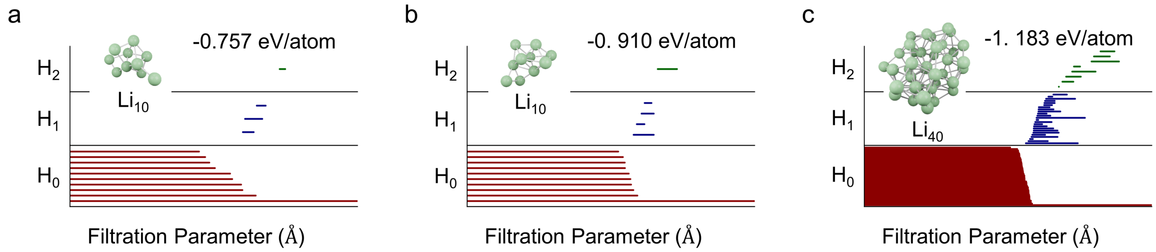

Supplementary Figure S3: Persistent homology analysis for three specific multi-atom systems. **a** and **b** show the topological fingerprints for two Li_{10} structures. The structure in **a** has the binding energies of -0.757 eV/atom. The binding energy of structure in **a** is -0.910 eV/atom. **c** shows the structure contains 40 atoms and has a richer topological information, and its binding energy is -1.183 eV/atom.

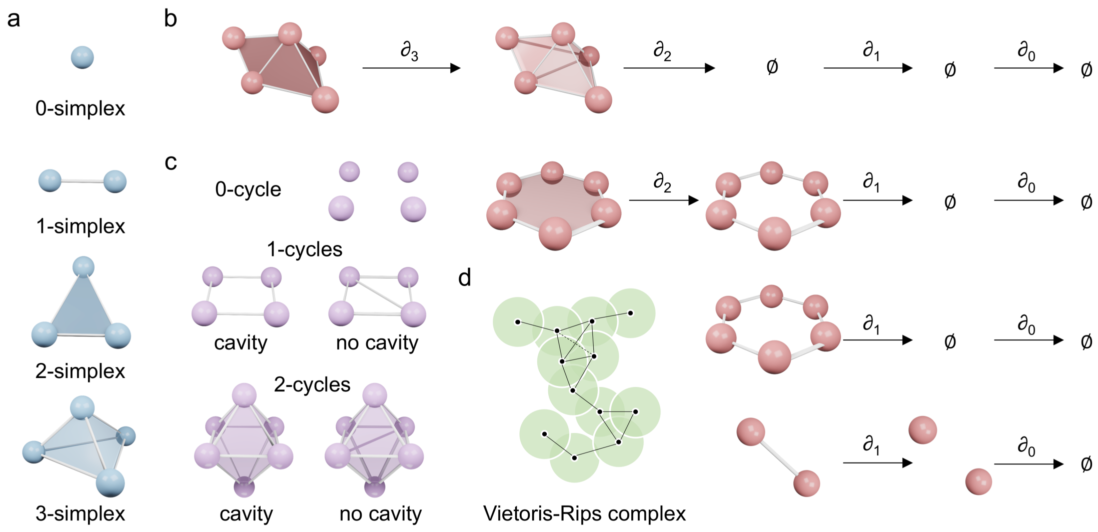

Supplementary Figure S4: Simplicial complex and boundary operator. **a** Illustration of 0-simples, 1-simplex, and 2-simplex. **b** Boundary maps take k-chains to their boundaries. The example shown in the figure (red sphere) is for dimensions 1 through 3. The empty set is denoted by $\emptyset$, and ∂_k with k=0, 1, 2, 3 represents the boundary operator of the corresponding dimension. **c** Illustration of k-cycles with k=0, 1, and 2 (purple shperes). For 1-cycles and 2-cycles, a trivial cycle (left) and a non-trivial (cavity-enclosing) cycle (right) are demonstrated. **d** Vietoris-Rips complex from a point cloud.

S3 Supplementary Note 3: Tables

Dataset The original dataset is generated from DFT calculations and it is derived from our previous work.[2] All structures and energies are contained in one file. For the file containing all data, each row contains the number of atoms, the coordinates, and the binding energy in eV per atom. The first number of each row is the number of atoms contained in the structure, the last data is the corresponding binding energy, and the rest of the data are the 3D coordinates of the atoms in the structure.

Supplementary Table S1: Statistic information of all multi-atom systems. Energy unit is eV/atom.

Datasets	Structures	Maximum Energy	Minimum Energy	Mean Energy	Median Energy
Li_4	8326	1.7337	-0.6567	-0.5258	-0.5734
Li_5	20988	2.1347	-0.7087	-0.5354	-0.6172
Li_6	20977	2.0881	-0.8346	-0.6275	-0.6962
Li_7	20998	2.0502	-0.9051	-0.6406	-0.7259
Li_8	21000	2.1364	-0.9462	-0.6739	-0.7552
Li_9	20999	1.4381	-0.9495	-0.6841	-0.7793
Li_{10}	20999	1.0743	-0.9927	-0.7089	-0.8059
Li_{20}	1000	-0.3215	-1.1052	-0.9084	-0.9488
Li_{40}	1000	-0.3905	-1.1832	-0.9541	-0.9899

Supplementary Table S2: Prediction results for Li_{20} and Li_{40} clusters by using L_0, L_{01}, L_{012}, L_1, L_2, and L_{12}

Tasks	Feature type	MAE	RMSE	PCC
Li20	L_0	0.079	0.084	0.982
Li20	L_{01}	0.139	0.145	0.968
Li20	L_{012}	0.174	0.182	0.944
Li20	L_1	0.293	0.302	0.925
Li20	L_2	0.650	0.667	0.762
Li20	L_{12}	0.267	0.277	0.899
Li40	L_0	0.119	0.126	0.968
Li40	L_{01}	0.27	0.274	0.954
Li40	L_{012}	0.302	0.308	0.925
Li40	L_1	0.689	0.707	0.922
Li40	L_2	0.905	0.921	0.891
Li40	L_{12}	0.578	0.606	0.894

Supplementary Table S3: Prediction results for Li_{20} and Li_{40} clusters by using L_0, β_{01}, β_{012}, β_1, β_2, and β_{12}

Tasks	Feature type	MAE	RMSE	PCC
Li20	β_0	0.139	0.158	0.508
Li20	β_{01}	0.118	0.134	0.742
Li20	β_{012}	0.113	0.126	0.771
Li20	β_1	0.122	0.145	0.603
Li20	β_2	0.185	0.212	0.451
Li20	β_{12}	0.117	0.138	0.611
Li40	β_0	0.203	0.221	0.592
Li40	β_{01}	0.18	0.192	0.801
Li40	β_{012}	0.168	0.179	0.817
Li40	β_1	0.134	0.158	0.35
Li40	β_2	0.195	0.219	0.555
Li40	β_{12}	0.125	0.152	0.385

Supplementary Table S4: Evaluation of five-fold cross-validation for Li_n clusters ($n = 4, 5, 6, 7, 8, 9, 10, 20, 40$).

Li_n cluster	Feature	MAE	RMSW	Pearson	Li_n cluster	Feature	MAE	RMSW	Pearson
4	β_0	0.034	0.045	0.975	9	β_0	0.044	0.055	0.98
4	β_{01}	0.031	0.045	0.978	9	β_{01}	0.037	0.045	0.985
4	β_{012}	0.031	0.045	0.978	9	β_{012}	0.036	0.045	0.985
4	β_1	0.108	0.205	0.058	9	β_1	0.152	0.245	0.489
4	β_2	0.11	0.205	0	9	β_2	0.185	0.277	0.141
4	β_{12}	0.108	0.205	0.058	9	β_{12}	0.15	0.243	0.497
5	β_0	0.035	0.045	0.983	10	β_0	0.041	0.055	0.981
5	β_{01}	0.033	0.045	0.985	10	β_{01}	0.034	0.045	0.987
5	β_{012}	0.033	0.045	0.985	10	β_{012}	0.034	0.045	0.987
5	β_1	0.142	0.247	0.248	10	β_1	0.145	0.23	0.547
5	β_2	0.151	0.253	0	10	β_2	0.185	0.27	0.166
5	β_{12}	0.142	0.247	0.248	10	β_{12}	0.143	0.228	0.558
6	β_0	0.044	0.055	0.974	20	β_0	0.031	0.045	0.96
6	β_{01}	0.039	0.055	0.978	20	β_{01}	0.019	0.032	0.985
6	β_{012}	0.04	0.055	0.978	20	β_{012}	0.017	0.032	0.988
6	β_1	0.142	0.239	0.266	20	β_1	0.04	0.055	0.922
6	β_2	0.152	0.247	0.027	20	β_2	0.091	0.126	0.553
6	β_{12}	0.142	0.239	0.267	20	β_{12}	0.037	0.055	0.93
7	β_0	0.045	0.055	0.976	40	β_0	0.028	0.032	0.976
7	β_{01}	0.039	0.055	0.98	40	β_{01}	0.019	0.032	0.988
7	β_{012}	0.039	0.055	0.98	40	β_{012}	0.017	0.032	0.99
7	β_1	0.154	0.247	0.355	40	β_1	0.037	0.055	0.937
7	β_2	0.172	0.265	0.077	40	β_2	0.073	0.11	0.733
7	β_{12}	0.153	0.247	0.356	40	β_{12}	0.033	0.055	0.948
8	β_0	0.052	0.063	0.972					
8	β_{01}	0.045	0.055	0.977					
8	β_{012}	0.044	0.055	0.978					
8	β_1	0.158	0.251	0.42					
8	β_2	0.181	0.274	0.133					
8	β_{12}	0.156	0.251	0.427					

References

[1] Fabian Pedregosa, Gaël Varoquaux, Alexandre Gramfort, Vincent Michel, Bertrand Thirion, Olivier Grisel, Mathieu Blondel, Peter Prettenhofer, Ron Weiss, Vincent Dubourg, et al. Scikit-learn: Machine learning in python. *the Journal of machine Learning research*, 12:2825–2830, 2011.

[2] Xin Chen, Dong Chen, Mouyi Weng, Yi Jiang, Guo-Wei Wei, and Feng Pan. Topology-based machine learning strategy for cluster structure prediction. *The journal of physical chemistry letters*, 11(11):4392–4401, 2020.
